# Supplementary material for: Implementation of an opioid use disorder (OUD) machine-learning phenotype in real-time for the ADAPT clinical trial
Source: PLOS Digit Health. 2026 Sep 3;5(9):e0001140. doi: 10.1371/journal.pdig.0001140 (PMC13541308; doi:10.1371/journal.pdig.0001140)
Supplement: Supplementary file 1 [file pdig.0001140.s001.docx]

**S1 Appendix.** Top 50 Feature Importance

| **Variable names** | **Importance** | **Category** |
| --- | --- | --- |
| Stimulant related disorders | 0.057985929 | PMH |
| Mental and substance use disorders in remission | 0.056217036 | PMH |
| Agents for opioid withdrawal opioid type | 0.04856072 | Medication |
| Other specified substance related disorders | 0.039214294 | PMH |
| Methadone presence in urine by screen method | 0.034579353 | Labs |
| Central alpha 2 receptor agonists | 0.034053132 | Medication |
| Antianxiety agent antihistamine type | 0.031491997 | Medication |
| Smoking deterrents nicotine type | 0.030542486 | Medication |
| Trauma and stressor related disorders | 0.030482968 | PMH |
| Opiates presence in urine by screen method | 0.030064206 | Labs |
| Psychological and psychiatric evaluation and therapy | 0.028295173 | Procedures |
| Diagnostic biologicals | 0.026110501 | Labs |
| Sedative related disorders | 0.022599257 | PMH |
| Vitamins B1 thiamine and derivatives | 0.022224854 | Medication |
| Cannabis related disorders | 0.018672519 | PMH |
| Depressive disorders | 0.017919428 | PMH |
| Alcohol related disorders | 0.01755891 | PMH |
| Opioid reversal agents opioid antagonists | 0.016917606 | Medication |
| Hepatitis | 0.016423581 | PMH |
| Poisoning by drugs initial encounter | 0.015698705 | PMH |
| Benzodiazepines presence in urine by screen method | 0.01466079 | Labs |
| Analgesic opioid agonists | 0.014398307 | Medication |
| Antidepressant serotonin 2 antagonist reuptake inhibitors saris | 0.013893774 | Medication |
| NSAID analgesics cox non specific propionic acid derivatives | 0.013297843 | Medication |
| Antianxiety agent benzodiazepines | 0.012715124 | Medication |
| Multivitamins | 0.01264001 | Medication |
| Vitamins folic acid and derivatives | 0.012363392 | Medication |
| Antidiarrheal antiperistaltic agents | 0.011786184 | Medication |
| Bipolar and related disorders | 0.011029416 | PMH |
| Disorders of teeth and gingiva | 0.009565986 | PMH |
| Unacceptable pdx* | 0.008979215 | Other* |
| Anticonvulsant gaba analogs | 0.008734985 | Medication |
| Suicidal ideationattemptintentional self harm | 0.007753035 | PMH |
| Antipsychotic atypical dopamine serotonin antag dibenzothiazepine der | 0.007724438 | Medication |
| Benzodiazepine sedative/hypnotic use | 0.007095145 | Medication |
| Other specified or unspecified mood disorder | 0.007008152 | PMH |
| Anxiety and fear related disorders | 0.006077084 | PMH |
| Saline or osmotic laxative use | 0.005765803 | Medication |
| Electrocardiogram | 0.005322162 | Procedures |
| Peripheral alpha 1 receptor blockers | 0.005291534 | Medication |
| Norepinephrine and dopamine reuptake inhibitor (NDRI) antidepressant use | 0.005046076 | Medication |
| Skin and subcutaneous tissue infections | 0.004903819 | PMH |
| Telehealth includes telephone calls online communication remote monitoring and surveillance | 0.00489416 | Procedures |
| Antidepressant selective serotonin reuptake inhibitors ssris | 0.004840033 | Medication |
| Alpha-2 receptor antagonist (NaSSA) antidepressant use | 0.004615419 | Medication |
| Antacid simethicone combinations | 0.004244199 | Medication |
| Skeletal muscle relaxant central muscle relaxants | 0.004191175 | Medication |
| Nicotinic receptor partial agonist (α4β2) smoking deterrent use | 0.003833983 | Medication |
| Low-molecular-weight heparin use | 0.003807975 | Medication |
| Other therapeutic procedures | 0.003765707 | Procedures |

* Diagnosis codes that are not appropriate as the principal diagnosis on a hospice claim or other Medicare claims
