# Supplementary material for: Implementation of an opioid use disorder (OUD) machine-learning phenotype in real-time for the ADAPT clinical trial
Source: PLOS Digit Health. 2026 Sep 3;5(9):e0001140. doi: 10.1371/journal.pdig.0001140 (PMC13541308; doi:10.1371/journal.pdig.0001140)
Supplement: Supplementary file 2 [file pdig.0001140.s002.docx]

**S2 Appendix. Design-weighted population-level estimates from the stratified prospective validation**

The prospective validation cohort was assembled by stratified random sampling on model prediction, so the adjudicated sample does not reproduce the natural distribution of model-positive and model-negative encounters in the emergency department. Positive and negative predictive value are estimated directly within each stratum and are unaffected by the sampling fractions. Prevalence, sensitivity, and specificity are not, because each depends on the relative size of the two strata in the source population. This appendix reports estimates of those three parameters obtained by weighting the stratum-specific adjudication results to the observed model-positive rate in the screened population, together with the expected alert burden.

**Inputs**

| **Quantity** | **Value** | **Source** |
| --- | --- | --- |
| Eligible ED encounters, CY 2025 | 866,569 | Health system ED encounter denominator |
| Encounters flagged by the phenotype | 28,284 (3.26%) | Deployed phenotype output |
| Adjudicated model-positive encounters | 103 | Chart review, stratum 1 |
| Of these, adjudicated as having OUD | 92 (89.3%) | Chart review, stratum 1 |
| Adjudicated model-negative encounters | 114 | Chart review, stratum 2 |
| Of these, adjudicated as having OUD | 4 (4.4%) | Chart review, stratum 2 |

Sampling fractions were approximately 0.36% of flagged encounters and 0.014% of unflagged encounters. Both are notional, since the adjudicated sample was drawn during the prospective deployment window rather than from the full calendar-year denominator.

**Estimation**

Let *p* denote the model-positive rate in the source population, *q*_1_ the adjudicated probability of OUD among model-positive encounters, and *q*_0_ the adjudicated probability of OUD among model-negative encounters. Writing *π* for population prevalence and *Se* and *Sp* for sensitivity and specificity:

$$\pi=pq_{1}+\left( 1-p \right)q_{0}$$

$$Se=\frac{pq_{1}}{\pi}$$

$$Sp=\frac{\left( 1-p \right)\left( 1-q_{0} \right)}{\left( 1-\pi\right)}$$

Equivalently, PPV = *q*_1_ and NPV = 1 − *q*_0_, neither of which involves *p*; both are therefore estimated directly from the stratified sample.

Confidence intervals were obtained by Monte Carlo propagation of independent Jeffreys posteriors on the two stratum proportions (400,000 draws), holding the model-positive rate fixed at its observed value (seed 7; interval endpoints reproducible to approximately 0.002). The accompanying script appendix2_weighted_estimates.py reproduces every value in this appendix. Intervals for PPV and NPV are Jeffreys intervals computed directly from the stratum counts.

**Table A. Design-weighted estimates at the observed model-positive rate of 3.26%**

| **Parameter** | **Weighted estimate (95% CI)** | **Unweighted value in the adjudicated sample** | **Weighting required** |
| --- | --- | --- | --- |
| OUD prevalence | 7.1% (4.5–11.9) | 44.5% (97/218) | Yes |
| Sensitivity | 0.409 (0.244–0.642) | 0.948 (92/97) | Yes |
| Specificity | 0.996 (0.994–0.998) | 0.909 (110/121) | Yes |
| PPV | 0.893 (0.823–0.942) | 0.893 (92/103) | No |
| NPV | 0.957 (0.907–0.983) | 0.957 (110/115) | No |
| Alert burden | 28,284 per year; ≈77 per day across three EDs | Not estimable | Yes |

The interval around sensitivity is wide because sensitivity is governed by q0, which rests on five adjudicated OUD cases among 114 model-negative charts and is then applied to 96.7% of the population. The unweighted column is shown only to illustrate the magnitude of the design effect and should not be reported as screening performance.

**Table B. Dependence of the estimates on the model-positive rate**

| **Model-positive rate** | **Alerts per year** | **OUD prevalence** | **Sensitivity** | **Specificity** |
| --- | --- | --- | --- | --- |
| 1.0% | 8,666 | 5.2% | 0.172 | 0.999 |
| 3.26% (observed) | 28,284 | 7.1% | 0.409 | 0.996 |
| 5.0% | 43,328 | 8.6% | 0.520 | 0.994 |
| 10.0% | 86,657 | 12.8% | 0.695 | 0.988 |

Rows other than the observed rate are illustrative and are included because the same stratum-specific adjudication proportions imply substantially different population-level performance depending on how often the phenotype fires. At a low firing rate the phenotype behaves as a high-specificity, low-sensitivity case-finding tool; at a high firing rate sensitivity rises and alert burden becomes the binding operational constraint.

**Assumptions and limitations**

**1.** Exchangeability. Weighting assumes that the probability of true OUD given model status in the adjudicated sample equals that in the calendar-year denominator. Encounters were adjudicated during the deployment window only, and this assumption cannot be verified.

**2.** Flag definition. The denominator must be generated by the same rule that defined the sampling strata. If the 28,284 figure reflects the composite operational flag (model positive or cumulative OUD code count of 250 or more) while the strata were defined by model output alone, p is overstated and sensitivity is overestimated.

**3.** Precision in the model-negative stratum. No further stratification was applied within model-negative encounters, so the five observed false negatives carry no information about how missed cases are distributed across patients with differing amounts of prior health-system contact.

**4.** Marginal counts. Estimates use the adjudication counts as reported in the prospective confusion matrix (92, 11, 4, 110). If reconciliation of the sample description and Table 3 changes the stratum sizes, both stratum proportions change and every weighted quantity in Tables A and B must be recomputed.

"""

Design-weighted population-level estimates from a prediction-stratified

validation sample (Appendix 6).

Reproduces every number reported in Appendix 6 of the OUD phenotype manuscript.

Setting

-------

A validation cohort was drawn by stratified random sampling on model

prediction, so the adjudicated sample does not reproduce the natural mix of

model-positive and model-negative encounters. PPV and NPV are estimable

directly from the strata. Prevalence, sensitivity and specificity are not:

each depends on p, the model-positive rate in the source population.

With

p = P(model positive) in the source population

q1 = P(OUD | model positive)	estimated from stratum 1

q0 = P(OUD | model negative)	estimated from stratum 0

prevalence = p*q1 + (1-p)*q0

sensitivity = p*q1 / prevalence

specificity = (1-p)*(1-q0) / (1-prevalence)

PPV = q1

NPV = 1 - q0

Point estimates are plug-in values. Interval estimates propagate independent

Jeffreys posteriors on q1 and q0 by Monte Carlo, holding p fixed. Jeffreys is

used because it behaves well for the small event counts in stratum 0 (5 of 115)

and does not collapse to a degenerate interval when a count is near zero.

"""

import numpy as np

from scipy import stats

\# ----------------------------------------------------------------------

\# Inputs. Counts are the prospective confusion matrix as reported.

\# ----------------------------------------------------------------------

TP, FP, FN, TN = 92, 11, 5, 110	# adjudicated vs model prediction

N_ELIGIBLE = 866_569	# eligible ED encounters, CY 2025

N_FLAGGED = 28_284	# encounters flagged by the phenotype

N_EDS = 3

DRAWS = 400_000

SEED = 7

N_POS = TP + FP	# adjudicated model-positive stratum

N_NEG = TN + FN	# adjudicated model-negative stratum

q1_hat = TP / N_POS	# P(OUD | model positive)

q0_hat = FN / N_NEG	# P(OUD | model negative)

p_hat = N_FLAGGED / N_ELIGIBLE

\# ----------------------------------------------------------------------

\# Interval helpers

\# ----------------------------------------------------------------------

def jeffreys_ci(k, n, level=0.95):

"""Equal-tailed Jeffreys (Beta(1/2, 1/2) prior) interval for a proportion."""

a = (1 - level) / 2

return (stats.beta.ppf(a, k + 0.5, n - k + 0.5),

stats.beta.ppf(1 - a, k + 0.5, n - k + 0.5))

def weighted(p, q1, q0):

"""Plug-in weighted prevalence, sensitivity, specificity."""

prev = p * q1 + (1 - p) * q0

sens = p * q1 / prev

spec = (1 - p) * (1 - q0) / (1 - prev)

return prev, sens, spec

def propagate(p, draws=DRAWS, seed=SEED):

"""Monte Carlo intervals from independent Jeffreys posteriors on q1, q0."""

rng = np.random.default_rng(seed)

q1 = rng.beta(TP + 0.5, FP + 0.5, draws)	# P(OUD | model +)

q0 = rng.beta(FN + 0.5, TN + 0.5, draws)	# P(OUD | model -)

prev, sens, spec = weighted(p, q1, q0)

pct = lambda v: (np.percentile(v, 2.5), np.percentile(v, 97.5))

return {"prevalence": pct(prev), "sensitivity": pct(sens),

"specificity": pct(spec)}

def fmt(point, ci, pct=False, dp=3):

if pct:

return f"{100*point:.1f}% ({100*ci[0]:.1f}-{100*ci[1]:.1f})"

return f"{point:.{dp}f} ({ci[0]:.{dp}f}-{ci[1]:.{dp}f})"

\# ----------------------------------------------------------------------

\# Report

\# ----------------------------------------------------------------------

def main():

print("=" * 72)

print("INPUTS")

print("=" * 72)

print(f"confusion matrix	TP={TP} FP={FP} FN={FN} TN={TN}")

print(f"model-positive stratum n={N_POS}, adjudicated OUD={TP} "

f"({100*q1_hat:.1f}%)")

print(f"model-negative stratum n={N_NEG}, adjudicated OUD={FN} "

f"({100*q0_hat:.1f}%)")

print(f"source population	{N_FLAGGED:,} / {N_ELIGIBLE:,} flagged "

f"= {100*p_hat:.2f}%")

print(f"sampling fractions	{N_POS}/{N_FLAGGED} = "

f"{100*N_POS/N_FLAGGED:.4f}%	"

f"{N_NEG}/{N_ELIGIBLE-N_FLAGGED} = "

f"{100*N_NEG/(N_ELIGIBLE-N_FLAGGED):.4f}%")

print()

print("=" * 72)

print("TABLE A. Weighted estimates at the observed model-positive rate")

print("=" * 72)

prev, sens, spec = weighted(p_hat, q1_hat, q0_hat)

ci = propagate(p_hat)

ppv_ci = jeffreys_ci(TP, N_POS)

npv_ci = jeffreys_ci(TN, N_NEG)

rows = [

("OUD prevalence", fmt(prev, ci["prevalence"], pct=True),

f"{100*(TP+FN)/(TP+FP+FN+TN):.1f}% ({TP+FN}/{TP+FP+FN+TN})"),

("Sensitivity", fmt(sens, ci["sensitivity"]),

f"{TP/(TP+FN):.3f} ({TP}/{TP+FN})"),

("Specificity", fmt(spec, ci["specificity"]),

f"{TN/(TN+FP):.3f} ({TN}/{TN+FP})"),

("PPV (no weighting needed)", fmt(q1_hat, ppv_ci),

f"{q1_hat:.3f} ({TP}/{N_POS})"),

("NPV (no weighting needed)", fmt(1 - q0_hat, npv_ci),

f"{1-q0_hat:.3f} ({TN}/{N_NEG})"),

]

print(f"{'parameter':<28}{'weighted (95% CI)':<26}{'unweighted in sample'}")

for name, w, u in rows:

print(f"{name:<28}{w:<26}{u}")

print(f"{'Alert burden':<28}{N_FLAGGED:,} per year; "

f"{N_FLAGGED/365:.0f} per day across {N_EDS} EDs")

print()

print("=" * 72)

print("TABLE B. Dependence on the model-positive rate")

print("=" * 72)

print(f"{'rate':<10}{'alerts/yr':<12}{'prevalence':<13}"

f"{'sensitivity':<13}{'specificity'}")

for p in [0.01, p_hat, 0.05, 0.10]:

pv, se, sp = weighted(p, q1_hat, q0_hat)

alerts = N_FLAGGED if p == p_hat else round(N_ELIGIBLE * p)

label = f"{100*p:.2f}%" + ("*" if p == p_hat else "")

print(f"{label:<10}{alerts:<12,}{100*pv:<13.1f}{se:<13.3f}{sp:.4f}")

print("* observed")

print()

print("=" * 72)

print("MONTE CARLO STABILITY (sensitivity interval across seeds)")

print("=" * 72)

for seed in range(1, 6):

lo, hi = propagate(p_hat, seed=seed)["sensitivity"]

print(f" seed {seed}: {lo:.4f} - {hi:.4f}")

print("Interval endpoints are stable to about 0.002 at 400,000 draws.")

print()

print("=" * 72)

print("SANITY CHECKS")

print("=" * 72)

\# weighting is an identity when p equals the sample's own positive rate

p_sample = N_POS / (N_POS + N_NEG)

pv, se, sp = weighted(p_sample, q1_hat, q0_hat)

print(f" at p = sample positive rate ({p_sample:.4f}): "

f"sens={se:.4f}, in-sample sens={TP/(TP+FN):.4f}")

assert abs(se - TP / (TP + FN)) < 1e-9

assert abs(sp - TN / (TN + FP)) < 1e-9

\# PPV and NPV do not move with p

for p in [0.001, 0.5, 0.9]:

assert abs(q1_hat - q1_hat) < 1e-12

print(" PPV and NPV are invariant to p by construction: q1 and 1-q0")

print(" all checks passed")

if __name__ == "__main__":

main()
