# Supplementary material for: Implementation of an opioid use disorder (OUD) machine-learning phenotype in real-time for the ADAPT clinical trial
Source: PLOS Digit Health. 2026 Sep 3;5(9):e0001140. doi: 10.1371/journal.pdig.0001140 (PMC13541308; doi:10.1371/journal.pdig.0001140)
Supplement: Supplementary file 3 [file pdig.0001140.s003.docx]

**S3 Appendix.** OUD ICD Code Value Set

| **ICD10 Code** | **Description** |
| --- | --- |
| F11.10 | Opioid abuse, uncomplicated |
| F11.120 | Opioid abuse with intoxication, uncomplicated |
| F11.121 | Opioid abuse with intoxication delirium |
| F11.122 | Opioid abuse with intoxication with perceptual disturbance |
| F11.129 | Opioid abuse with intoxication, unspecified |
| F11.14 | Opioid abuse with opioid-induced mood disorder |
| F11.150 | Opioid abuse with opioid-induced psychotic disorder with delusions |
| F11.151 | Opioid abuse with opioid-induced psychotic disorder with hallucinations |
| F11.159 | Opioid abuse with opioid-induced psychotic disorder, unspecified |
| F11.181 | Opioid abuse with opioid-induced sexual dysfunction |
| F11.182 | Opioid abuse with opioid-induced sleep disorder |
| F11.188 | Opioid abuse with other opioid-induced disorder |
| F11.19 | Opioid abuse with unspecified opioid-induced disorder |
| F11.20 | Opioid dependence, uncomplicated |
| F11.21 | Opioid dependence, in remission |
| F11.220 | Opioid dependence with intoxication, uncomplicated |
| F11.221 | Opioid dependence with intoxication delirium |
| F11.222 | Opioid dependence with intoxication with perceptual disturbance |
| F11.229 | Opioid dependence with intoxication, unspecified |
| F11.23 | Opioid dependence with withdrawal |
| F11.24 | Opioid dependence with opioid-induced mood disorder |
| F11.250 | Opioid dependence with opioid-induced psychotic disorder with delusions |
| F11.251 | Opioid dependence with opioid-induced psychotic disorder with hallucinations |
| F11.259 | Opioid dependence with opioid-induced psychotic disorder, unspecified |
| F11.281 | Opioid dependence with opioid-induced sexual dysfunction |
| F11.282 | Opioid dependence with opioid-induced sleep disorder |
| F11.288 | Opioid dependence with other opioid-induced disorder |
| F11.29 | Opioid dependence with unspecified opioid-induced disorder |
| F11.90 | Opioid use, unspecified, uncomplicated |
| F11.920 | Opioid use, unspecified with intoxication, uncomplicated |
| F11.921 | Opioid use, unspecified with intoxication delirium |
| F11.922 | Opioid use, unspecified with intoxication with perceptual disturbance |
| F11.929 | Opioid use, unspecified with intoxication, unspecified |
| F11.93 | Opioid use, unspecified with withdrawal |
| F11.94 | Opioid use, unspecified with opioid-induced mood disorder |
| F11.950 | Opioid use, unspecified with opioid-induced psychotic disorder with delusions |
| F11.951 | Opioid use, unspecified with opioid-induced psychotic disorder with hallucinations |
| F11.959 | Opioid use, unspecified with opioid-induced psychotic disorder, unspecified |
| F11.981 | Opioid use, unspecified with opioid-induced sexual dysfunction |
| F11.982 | Opioid use, unspecified with opioid-induced sleep disorder |
| F11.988 | Opioid use, unspecified with other opioid-induced disorder |
| F11.99 | Opioid use, unspecified with unspecified opioid-induced disorder |
| T40.0X1A | Poisoning by opium, accidental (unintentional), initial encounter |
| T40.1X1A | Poisoning by heroin, accidental (unintentional), initial encounter |
| T40.2X1A | Poisoning by other opioids (e.g., morphine, codeine), accidental (unintentional), initial encounter |
| T40.3X1A | Poisoning by methadone, accidental (unintentional), initial encounter |
| T40.4X1A | Poisoning by other synthetic narcotics (e.g., fentanyl, tramadol), accidental (unintentional), initial encounter |
| T40.601A | Poisoning by unspecified narcotics, accidental (unintentional), initial encounter |
| T40.0X2A | Poisoning by opium, intentional self-harm, initial encounter |
| T40.1X2A | Poisoning by heroin, intentional self-harm, initial encounter |
| T40.2X2A | Poisoning by other opioids, intentional self-harm, initial encounter |
| T40.3X2A | Poisoning by methadone, intentional self-harm, initial encounter |
| T40.4X2A | Poisoning by other synthetic narcotics, intentional self-harm, initial encounter |
| T40.601D | Poisoning by unspecified narcotics, accidental (unintentional), subsequent encounter |
| T40.2X5A | Adverse effect of other opioids, initial encounter |
| T40.3X5A | Adverse effect of methadone, initial encounter |
| T40.4X5A | Adverse effect of other synthetic narcotics, initial encounter |
| T40.605A | Adverse effect of unspecified narcotics, initial encounter |
