# Supplementary material for: Implementation of an opioid use disorder (OUD) machine-learning phenotype in real-time for the ADAPT clinical trial
Source: PLOS Digit Health. 2026 Sep 3;5(9):e0001140. doi: 10.1371/journal.pdig.0001140 (PMC13541308; doi:10.1371/journal.pdig.0001140)
Supplement: Supplementary file 4 [file pdig.0001140.s004.docx]

**S4 Appendix. OUD Counts per Patient per Day Summary Statistics**

| Mean | Std | Min | 25% | 50% | 75% | Max |
| --- | --- | --- | --- | --- | --- | --- |
| 0.043 | 0.421 | 0.000 | 0.000 | 0.000 | 0.000 | 26.000 |
