# Supplementary material for: Implementation of an opioid use disorder (OUD) machine-learning phenotype in real-time for the ADAPT clinical trial
Source: PLOS Digit Health. 2026 Sep 3;5(9):e0001140. doi: 10.1371/journal.pdig.0001140 (PMC13541308; doi:10.1371/journal.pdig.0001140)
Supplement: Supplementary file 5 [file pdig.0001140.s005.docx]

**S5 Appendix.** Chart Review Guidelines

**Overall goal:** Using EHR data present **at the start of the index ED encounter**, identify, to the best of your ability, whether each patient meets the DSM-5 definition of opioid use disorder (below). In ambiguous cases, you may have to use your judgement.

**Approach:**

**A.** Review **structured data elements** (e.g., problem lists, medication lists, urine toxicology results). [Note that if you hover your cursor over a diagnosis or medication, you can find date information to confirm that items preceded the index ED visit.]

**B.** Review the **text of clinical notes**. In reviewing clinical notes, please take two approaches:

1.	Review (if present) the patient’s most recent notes of the following types *prior to* the index ED encounter:

a.	hospital discharge summary

b.	ED provider note

c.	Addiction psychiatry note [Chart Review Notes Filters Author Specialty Addiction psychiatry]

2.	Using Epic’s search function, search for text related to the DSM-5 criteria below. Recommended search terms, adapted from Palumbo et al., (doi:10.1001/jamanetworkopen.2020.15909), are presented in blue text below each DSM-5 item. Note that searches may return many results unrelated to patients’ opioid use; in making the OUD diagnosis, however, you should only consider opioid-related symptoms or circumstances.

**DSM-5 definition of opioid use disorder:**

A problematic pattern of opioid use leading to clinically significant impairment or distress, as manifested by **at least two** of the following, occurring **within a 12-month period**:

1. Opioids are often taken in larger amounts or over a longer period than was intended.
2. There is a persistent desire or unsuccessful efforts to cut down or control opioid use.

**SEARCH TERMS:**

a.	Cut down

b.	Cut back

c.	Wean

d.	Reduce

3. A great deal of time is spent in activities necessary to obtain the opioid, use the opioid, or recover from its effects.
4. Craving, or a strong desire or urge to use opioids.

**SEARCH TERMS:**

a.	Craving

b.	Urge

c.	Dependence

5. Recurrent opioid use resulting in a failure to fulfill major role obligations at work, school, or home.

**SEARCH TERMS:**

a.	Occupation

b.	Job

c.	Boss

6. Continued opioid use despite having persistent or recurrent social or interpersonal problems caused or exacerbated by the effects of opioids.

**SEARCH TERMS:**

a.	Lawyer

b.	Legal

c.	Jail

d.	Prison

e.	Charges

7. Important social, occupational, or recreational activities are given up or reduced because of opioid use.
8. Recurrent opioid use in situations in which it is physically hazardous.

**SEARCH TERMS:**

a.	Overdose

b.	Fall

c.	Driving

9. Continued opioid use despite knowledge of having a persistent or recurrent physical or psychological problem that is likely to have been caused or exacerbated by the substance.

**SEARCH TERMS:**

a.	Constipation

b.	Hepatitis C

c.	HIV

d.	Abscess

e.	Substance-induced

10. Tolerance, as defined by either of the following:
    1. A need for markedly increased amounts of opioids to achieve intoxication or desired effect.
    1. A markedly diminished effect with continued use of the same amount of an opioid.

**Note:** This criterion is not considered to be met for those taking opioids solely under appropriate medical supervision.

**SEARCH TERMS:**

a.	Tolerance

11. Withdrawal, as manifested by either of the following:
    1. The characteristic opioid withdrawal syndrome (refer to Criteria A and B of the criteria set for opioid withdrawal, pp. 547–548).
    2. Opioids (or a closely related substance) are taken to relieve or avoid withdrawal symptoms.

**Note:** This criterion is not considered to be met for those individuals taking opioids solely under appropriate medical supervision.

**SEARCH TERMS:**

**a.**	Withdrawal
