# Supplementary material for: Implementation of an opioid use disorder (OUD) machine-learning phenotype in real-time for the ADAPT clinical trial
Source: PLOS Digit Health. 2026 Sep 3;5(9):e0001140. doi: 10.1371/journal.pdig.0001140 (PMC13541308; doi:10.1371/journal.pdig.0001140)
Supplement: Supplementary file 6 [file pdig.0001140.s007.docx]

**S7 Appendix.** Prescribed Medications List

| MEDICATION_ID | MEDICATION_NAME |
| --- | --- |
| 1226 | BUPRENEX 0.3 MG/ML INJECTION SOLUTION |
| 4950 | METHADONE INTENSOL 10 MG/ML ORAL CONCENTRATE |
| 4951 | METHADONE 10 MG/5 ML ORAL SOLUTION |
| 4952 | METHADONE 5 MG/5 ML ORAL SOLUTION |
| 4953 | METHADONE 10 MG TABLET |
| 4954 | METHADONE 5 MG TABLET |
| 4956 | METHADOSE 10 MG/ML ORAL CONCENTRATE |
| 9320 | BUPRENORPHINE HCL 0.3 MG/ML INJECTION SYRINGE |
| 10685 | NALTREXONE 50 MG TABLET |
| 15996 | METHADONE 10 MG/ML ORAL CONCENTRATE |
| 16182 | METHADOSE 40 MG SOLUBLE TABLET |
| 23563 | NALTREXONE HCL (BULK) 100 % POWDER |
| 34711 | BUPRENORPHINE HCL 2 MG SUBLINGUAL TABLET |
| 34712 | BUPRENORPHINE HCL 8 MG SUBLINGUAL TABLET |
| 34713 | BUPRENORPHINE 2 MG-NALOXONE 0.5 MG SUBLINGUAL TABLET |
| 34714 | BUPRENORPHINE 8 MG-NALOXONE 2 MG SUBLINGUAL TABLET |
| 41938 | METHADONE 40 MG SOLUBLE TABLET |
| 42251 | NALTREXONE (BULK) 100 % POWDER |
| 76527 | NALTREXONE ER 380 MG INTRAMUSCULAR SUSPENSION,EXTENDED RELEASE |
| 76530 | VIVITROL 380 MG INTRAMUSCULAR SUSPENSION,EXTENDED RELEASE |
| 113429 | BUPRENORPHINE HCL 0.3 MG/ML INJECTION SOLUTION |
| 116665 | DISKETS 40 MG SOLUBLE TABLET |
| 118411 | METHADONE HCL |
| 118418 | BUPRENORPHINE HCL |
| 118474 | NALTREXONE HCL |
| 122401 | BUPRENORPHINE HCL/NALOXONE HCL |
| 122949 | NALTREXONE |
| 123066 | NALTREXONE MICROSPHERES |
| 127797 | BUPRENEX INJ |
| 127801 | BUPRENORPHINE-NALOXONE SL |
| 130608 | DEPADE ORAL |
| 131284 | DISKETS ORAL |
| 131376 | DOLOPHINE ORAL |
| 137382 | METHADONE INTENSOL ORAL |
| 137383 | METHADONE ORAL |
| 137384 | METHADOSE ORAL |
| 138377 | NALTREXONE (BULK) MISC |
| 138378 | NALTREXONE HCL (BULK) MISC |
| 138379 | NALTREXONE MICROSPHERES IM |
| 138381 | NALTREXONE ORAL |
| 142735 | REVIA ORAL |
| 144306 | SUBOXONE SL |
| 144307 | SUBUTEX SL |
| 146993 | VIVITROL IM |
| 150675 | METHADONE 10 MG/ML ORAL SYRINGE (FOR ORAL USE ONLY) |
| 155055 | BUPRENORPHINE 4 MG-NALOXONE 1 MG SUBLINGUAL FILM |
| 155071 | BUPRENORPHINE 12 MG-NALOXONE 3 MG SUBLINGUAL FILM |
| 155087 | SUBOXONE 4 MG-1 MG SUBLINGUAL FILM |
| 155088 | SUBOXONE 12 MG-3 MG SUBLINGUAL FILM |
| 158927 | BUPRENORPHINE 1.4 MG-NALOXONE 0.36 MG SUBLINGUAL TABLET |
| 158931 | BUPRENORPHINE 5.7 MG-NALOXONE 1.4 MG SUBLINGUAL TABLET |
| 158999 | ZUBSOLV 1.4 MG-0.36 MG SUBLINGUAL TABLET |
| 159000 | ZUBSOLV 5.7 MG-1.4 MG SUBLINGUAL TABLET |
| 159320 | BUPRENORPHINE 15 MCG/HOUR WEEKLY TRANSDERMAL PATCH |
| 159326 | BUTRANS 15 MCG/HOUR TRANSDERMAL PATCH |
| 160248 | ZUBSOLV SL |
| 161155 | BUPRENORPHINE 2 MG-NALOXONE 0.5 MG SUBLINGUAL FILM |
| 161156 | BUPRENORPHINE 8 MG-NALOXONE 2 MG SUBLINGUAL FILM |
| 161161 | SUBOXONE 2 MG-0.5 MG SUBLINGUAL FILM |
| 161162 | SUBOXONE 8 MG-2 MG SUBLINGUAL FILM |
| 161686 | BUPRENORPHINE 5 MCG/HOUR WEEKLY TRANSDERMAL PATCH |
| 161687 | BUPRENORPHINE 10 MCG/HOUR WEEKLY TRANSDERMAL PATCH |
| 161688 | BUPRENORPHINE 20 MCG/HOUR WEEKLY TRANSDERMAL PATCH |
| 161846 | BUTRANS 5 MCG/HOUR TRANSDERMAL PATCH |
| 161847 | BUTRANS 10 MCG/HOUR TRANSDERMAL PATCH |
| 161848 | BUTRANS 20 MCG/HOUR TRANSDERMAL PATCH |
| 163308 | BUPRENORPHINE TD |
| 163309 | BUTRANS TD |
| 165145 | BUPRENORPHINE |
| 171408 | NALTREXONE HCL DIHYDRATE (BULK) 100 % POWDER |
| 171744 | BUPRENORPHINE 7.5 MCG/HOUR WEEKLY TRANSDERMAL PATCH |
| 171976 | BUTRANS 7.5 MCG/HOUR TRANSDERMAL PATCH |
| 172165 | BUNAVAIL BUCL |
| 172167 | BUPRENORPHINE HCL INJ |
| 172168 | BUPRENORPHINE HCL SL |
| 172169 | BUPRENORPHINE-NALOXONE BUCL |
| 172490 | NALTREXONE HCL DIHYDRATE(BULK) MISC |
| 172861 | NALTREXONE HCL DIHYDRATE |
| 174318 | BUPRENORPHINE 8.6 MG-NALOXONE 2.1 MG SUBLINGUAL TABLET |
| 174324 | ZUBSOLV 8.6 MG-2.1 MG SUBLINGUAL TABLET |
| 176621 | BUPRENORPHINE 11.4 MG-NALOXONE 2.9 MG SUBLINGUAL TABLET |
| 176830 | BUPRENORPHINE 2.9 MG-NALOXONE 0.71 MG SUBLINGUAL TABLET |
| 176833 | ZUBSOLV 11.4 MG-2.9 MG SUBLINGUAL TABLET |
| 176835 | ZUBSOLV 2.9 MG-0.71 MG SUBLINGUAL TABLET |
| 177142 | BUPRENORPHINE HCL 75 MCG BUCCAL FILM |
| 177143 | BUPRENORPHINE HCL 150 MCG BUCCAL FILM |
| 177144 | BUPRENORPHINE HCL 300 MCG BUCCAL FILM |
| 177145 | BUPRENORPHINE HCL 450 MCG BUCCAL FILM |
| 177146 | BUPRENORPHINE HCL 600 MCG BUCCAL FILM |
| 177147 | BUPRENORPHINE HCL 750 MCG BUCCAL FILM |
| 177148 | BUPRENORPHINE HCL 900 MCG BUCCAL FILM |
| 177173 | BELBUCA 75 MCG BUCCAL FILM |
| 177174 | BELBUCA 150 MCG BUCCAL FILM |
| 177175 | BELBUCA 300 MCG BUCCAL FILM |
| 177176 | BELBUCA 450 MCG BUCCAL FILM |
| 177177 | BELBUCA 600 MCG BUCCAL FILM |
| 177178 | BELBUCA 750 MCG BUCCAL FILM |
| 177179 | BELBUCA 900 MCG BUCCAL FILM |
| 177421 | BELBUCA BUCL |
| 177429 | BUPRENORPHINE HCL BUCL |
| 181027 | BUPRENORPHINE HCL SDRM |
| 181165 | PROBUPHINE SDRM |
| 182702 | BUPRENORPHINE 0.7 MG-NALOXONE 0.18 MG SUBLINGUAL TABLET |
| 182703 | ZUBSOLV 0.7 MG-0.18 MG SUBLINGUAL TABLET |
| 186317 | BUPRENORPHINE ER 300 MG/1.5 ML SOLUTION,EXTEN.REL.SUBCUTANEOUS SYRINGE |
| 186318 | BUPRENORPHINE ER 100 MG/0.5 ML SOLUTION,EXTEN.REL.SUBCUTANEOUS SYRINGE |
| 186362 | SUBLOCADE 100 MG/0.5 ML SOLUTION,EXTENDED RELEASE SUBCUTANEOUS SYRINGE |
| 186363 | SUBLOCADE 300 MG/1.5 ML SOLUTION,EXTENDED RELEASE SUBCUTANEOUS SYRINGE |
| 186378 | BUPRENORPHINE SUBQ |
| 186426 | SUBLOCADE SUBQ |
| 198418 | METHADONE 5 MG/0.5 ML ORAL SYRINGE (FOR ORAL USE ONLY) |
| 206631 | NALTREXONE 4.5 MG CAPSULE |
| 206649 | NALTREX 4.5 MG CAPSULE |
| 206683 | NALTREX ORAL |
| 206693 | BUPRENORPHINE ER 8 MG/0.16 ML SOLUTION,EXTEN.REL.SUBCUTANEOUS SYRINGE WEEKLY |
| 206694 | BUPRENORPHINE ER 16 MG/0.32 ML SOLUTION,EXTEN.REL.SUBCUTANEOUS SYRINGE WEEKLY |
| 206695 | BUPRENORPHINE ER 24 MG/0.48 ML SOLUTION,EXTEN.REL.SUBCUTANEOUS SYRINGE WEEKLY |
| 206696 | BUPRENORPHINE ER 32 MG/0.64 ML SOLUTION,EXTEN.REL.SUBCUTANEOUS SYRINGE WEEKLY |
| 206697 | BUPRENORPHINE ER 64 MG/0.18 ML SOLUTION,EXTEN.REL.SUBCUTANEOUS SYRINGE MONTHLY |
| 206698 | BUPRENORPHINE ER 96 MG/0.27 ML SOLUTION,EXTEN.REL.SUBCUTANEOUS SYRINGE MONTHLY |
| 206699 | BUPRENORPHINE ER 128 MG/0.36 ML SOLUTION,EXT.REL.SUBCUTANEOUS SYRINGE MONTHLY |
| 206714 | BRIXADI MONTHLY 128 MG/0.36 ML SOLUTION,EXTEND.REL. SUBCUT.SYRINGE |
| 206715 | BRIXADI WEEKLY 8 MG/0.16 ML SOLUTION,EXTEND.REL. SUBCUT.SYRINGE |
| 206716 | BRIXADI WEEKLY 16 MG/0.32 ML SOLUTION,EXTEND.REL. SUBCUT.SYRINGE |
| 206717 | BRIXADI WEEKLY 24 MG/0.48 ML SOLUTION,EXTEND.REL. SUBCUT.SYRINGE |
| 206718 | BRIXADI WEEKLY 32 MG/0.64 ML SOLUTION,EXTEND.REL. SUBCUT.SYRINGE |
| 206719 | BRIXADI MONTHLY 64 MG/0.18 ML SOLUTION,EXTEND.REL. SUBCUT.SYRINGE |
| 206720 | BRIXADI MONTHLY 96 MG/0.27 ML SOLUTION,EXTEND.REL. SUBCUT.SYRINGE |
| 206749 | BRIXADI SUBQ |
| 207457 | LOTREXONE 4.5 MG CAPSULE |
| 207458 | NALTREXONE 1.5 MG CAPSULE |
| 207464 | LOTREXONE 1.5 MG CAPSULE |
| 207497 | LOTREXONE ORAL |
| 207837 | NALTREX 1.5 MG CAPSULE |
| 210465 | NALTREXONE HCL, MICRONIZED (BULK) 100 % POWDER |
| 210524 | NALTREXONE HCL,MICRONIZE(BULK) MISC |
| 210541 | NALTREXONE HCL,MICRONIZED |
| 401824 | BUPRENORPHINE (SUBUTEX) 1 MG SUBLINGUAL HALFTAB |
| 401836 | BUPRENORPHINE (SUBUTEX) 4 MG SUBLINGUAL HALFTAB |
| 426007 | NALTREXONE (REVIA) 50 MG HALFTAB |
| 453007 | METHADONE 2.5 MG HALFTAB |
| 40815296 | NALTREXONE ER 380 MG INTRAMUSCULAR SUSPENSION FREE DRUG PROGRAM |
| 40815418 | METHADONE 5 MG TABLET (WRAPPED E-RX) |
